# Relations among obesity, family socioeconomic status, oral health behaviors, and dental caries in adolescents: the 2010–2012 Korea National Health and nutrition examination survey

**DOI:** 10.1186/s12903-018-0576-5

**Published:** 2018-06-22

**Authors:** Jin Ah Kim, Hayon Michelle Choi, Yunhee Seo, Dae Ryong Kang

**Affiliations:** 1 College of Nursing Science, Kyung Hee University, Seoul, Korea; 2 Graduate School of Public Health, Seoul National University, Seoul, Korea; 3 Graduate School of Public Health, Ajou University, Suwon, Korea; 4 Center of Biomedical Data Science / Institute of Genomic Cohort, Wonju College of Medicine, Yonsei University, 20 Ilsan-ro, Wonju, Gangwon-do 26426 Korea

**Keywords:** Dental caries, Adolescent, Oral health, Cross-sectional study

## Abstract

**Background:**

The purpose of this study was to examine the relationships among obesity, family socioeconomic status, oral health behaviors, and dental caries and to identify possible differences in factors related with dental caries according to gender among a representative sample of Korean adolescents.

**Methods:**

Data were obtained from the Korean National Health and Nutrition Examination Survey, which was conducted between 2010 and 2012. This nationally representative cross-sectional survey included approximately 10,000 individuals, including adolescents, each year as a survey sample, and collected information on socioeconomic status, health-related behaviors, quality of life, healthcare utilization, anthropometric measures, biochemical and clinical profiles for non-communicable diseases, and dietary intake via three component surveys (health interview, health examination, and nutrition survey). The health interview and health examination were conducted by trained staff members. A total of 1646 adolescents of ages 13 to 18 years old were included in this study; there were 879 males and 767 females. Data were analyzed by t-test, X^2^-test, and univariate and multivariate logistic regression analyses using SAS 9.4 and ‘R’ statistical software for Windows to account for the complex sampling design.

**Results:**

In males, significant associations between family income and dental caries on permanent teeth were noted after adjusting for confounding variables; the odds ratios and 95% confidence intervals thereof were 0.43(0.24–0.76), 0.41(0.24–0.70), and 0.28(0.16–0.49) for low-middle, middle-high, and high family income, respectively. Smoking experience showed a significant association with dental caries on permanent teeth in females. Oral health behaviors, such as tooth brushing frequency, were associated with dental caries in only male adolescents. There was no association between obesity and dental caries on permanent teeth in either male or female adolescents.

**Conclusion:**

The present study demonstrated that factors associated with dental caries in adolescents differ according to gender. Therefore, gender-specific interventions may be warranted to improve dental health among adolescents.

## Background

Rapid development of modern society and improvement in income status are affecting human health, particularly obesity rates, which have doubled over the past 30 years [1]. Obesity rates in adolescents have been increasing steadily since the 1980s [1]. A serious concern, adolescent obesity has been shown to be closely related to risk factors for chronic diseases, such as hyperlipidemia, hypertension, diabetes, and fatty liver, in adulthood [2, 3]. Furthermore, previous studies have shown that obesity is significantly correlated with dental caries on permanent teeth in adults and adolescents [4, 5]. According to the national statistics of Korea, the incidence rate of dental caries in Korean adolescents is 38%, with a decayed, missing, and filled teeth index (DMFT) score of 1.91, which is higher than the average score of 1.6 among Organization for Economic Cooperation and Development countries. Additionally, statistics indicate that one third of all dental patients in Korea are adolescents [6]. Considering the high rate of obesity and prevalence of dental caries in adolescents, we suspect that there may be a relationship between obesity and dental caries. In addition to obesity, family socioeconomic status may be a risk factor for dental caries in adolescents. Among the factors affecting dental caries in adults, socioeconomic status could be the major risk factor. Previous studies on adults have reported that individuals from lower socioeconomic status tend to exhibit a higher number of risk factors for dental caries than those of higher socioeconomic status [7]. Based on these prior studies, it can be inferred that family economic status can affect dental caries in adolescence.

Building healthy dental care habits in adolescence is important, since dental health at this time is related to dental health throughout one’s entire life. A previous study showed that women have better oral hygiene habits than men [8]. Therefore, identifying possible differences in oral health behaviors in relation to dental caries on permanent teeth according to gender can help with developing oral health policies through which to prevent dental caries on permanent dental caries.

Although it is important to confirm the relationships among obesity, family socioeconomic status, oral health behaviors, and dental caries in adolescents, studies on dental caries in adolescents in relation to these factors are limited in Korea. Herein, the hypothesis of this study was that obesity, family socioeconomic status, and oral health behaviors would be related to dental caries on permanent teeth in adolescents. Therefore, the present study was performed to identify associations among obesity, socioeconomic status, oral health behaviors, and dental caries and to find out possible differences in factors related with dental caries according to gender among adolescents in a large sample from the Korean population using data from the Korea National Health and Nutrition Examination Survey (KNHANES).

## Methods

### Data collection and measurement

The Korea National Health and Nutrition Examination Survey (KNHANES) is a national surveillance system that has been assessing the health and nutritional status of Koreans since 1998. Based on the National Health Promotion Act, the surveys have been conducted by the Korea Centers for Disease Control and Prevention (KCDCP). This nationally representative cross-sectional survey includes approximately 10,000 individuals each year as a survey sample, and collects information on socioeconomic status, health-related behaviors, quality of life, healthcare utilization, anthropometric measures, biochemical and clinical profiles for non-communicable diseases, and dietary intakes with three component surveys: health interview, health examination and nutrition survey. The health interview and health examination are conducted by trained staff members, including physicians, dentists, medical technicians, and health interviewers, at a mobile examination center, and dieticians who visit the homes of the study participants for follow up. This study includes data from the KNHANES between 2010 and 2012. In total, 1829 individuals aged 13–18 years were targeted for the survey, and 1646 participants with complete data were evaluated in the present study.

#### Demographic and socioeconomic variables

In the present study, demographic and socioeconomic status were assessed according to age, gender, alcohol experience, smoking experience, monthly household income, and residential area. Monthly household income levels were divided into quartiles in consideration of the number of family members. Based on the results, the first group was defined as the lowest income group and the fourth group as the highest income group: specifically, quartiles comprised 10 thousand ~ 1 million won, 1.01–2 million won, 2.01–2.8 million won, and 2.81–5 million won or more. Sixteen living areas were categorized as rural and urban.

#### Oral health behaviors

Experience of a dental checkup within a year and whether adolescents brushed their teeth regularly after every meal (breakfast, lunch, and dinner) were assessed.

#### Anthropometric measurements

Body weight and height were measured to the nearest 0.1 kg and 0.1 cm, respectively, whilst participants were wearing light indoor clothing without shoes. Body mass index (BMI) was calculated using the following formula: BMI = body weight (kg)/height^2^ (m^2^). In general, the BMI (kg/m^2^) cut-off points for overweight and obese are set at 25 and 30, respectively, in adults. However, in children and adolescents, “overweight” is defined as a BMI (kg/m^2^) ≥ the sex–age-specific 95th BMI percentile and “at risk for overweight” as 85th ≤ BMI <95th percentile [9, 10]. Based on these references, we defined “obesity” as a BMI (kg/m^2^) ≥ the sex–age-specific 95th BMI percentile.

### Data analysis

Data were analyzed by t-test, X^2^-test, and univariate and multivariate logistic regression analysis using the SAS statistical software package for Windows (version 9.4) to account for the complex sampling design. Two-sided *p*-values < 0.05 were considered to indicate a statistically significant difference, and weight estimates were also considered.

## Results

Table 1 shows the general characteristics, family socioeconomic status, and oral health behaviors of the study population divided according to gender. The age of the male participants who exhibited permanent tooth decay was 15.81 ± 0.11 years old, while that of male participants who did not was 15.47 ± 0.08 years old (*p*-value = 0.011). The incident rates of permanent tooth decay were higher in both males and females of low income status than those of high income status (*p*-value< .001). Drinking experience was significantly more common in male adolescents with permanent decay that in those without permanent decay; this was not observed in females (Male: *p*-value = 0.010; Female: *p*-value = 0.206). Smoking experience, however, was more common in both male and female adolescents with permanent decay than those without (Male: *p*-value = 0.004; Female: *p*-value < .001).

**Table 1:** Characteristics of the study population

	Incidence of permanent tooth decay					
Variables	Male			Female		
	No	Yes	*p*-value^§^	No	Yes	*p*-value^§^
Age (years) ^†^	15.47 ± 0.08	15.81 ± 0.11	0.011	15.50 ± 0.08	15.59 ± 0.14	0.603^‡^
Income (quartiles)						
Q1 (lowest)	56 (9.16)	49 (19.44)	<.001	64 (11.19)	40 (21.86	<.001
Q2	140 (22.91)	67 (26.58)		142 (24.83)	58 (31.69)	
Q3	179 (29.29)	74 (29.36)		176 (30.77)	50 (27.32)	
Q4 (highest)	236 (38.62)	62 (24.60)		190 (33.22)	35 (19.13)	
Obesity						
Normal	528 (84.75)	212 (82.81)	0.500	523 (89.86)	160 (86.49)	0.118
Obesity	95 (15.25)	44 (17.19)		59 (10.14)	25 (13.51)	
Living area						
Rural	336 (53.93)	135 (52.73)	0.536	323 (55.50)	105 (56.76)	0.745
Urban	287 (46.07)	121 (47.27)		259 (44.50)	80 (43.24)	
Drinking experience						
No	403 (65.32)	141 (55.29)	0.010	388 (67.36)	113 (61.41)	0.206
Yes	214 (34.68)	114 (44.71)		188 (32.64)	71 (38.59)	
Smoking experience						
No	489 (79.25)	170 (66.93)	0.004	524 (90.97)	153 (83.15)	<.001
Yes	128 (20.75)	84 (33.07)		52 (9.03)	31 (16.85)	
Dental checkup within a year						
No	400 (64.62)	187 (73.33)	0.001	321 (55.44)	119 (64.32)	0.103
Yes	219 (35.38)	68 (26.67)		258 (44.56)	66 (35.68)	
Tooth brushing after every meal						
No	430 (70.382)	194 (76.98)	0.029	281 (48.78)	94(51.09)	0.315
Yes	181 (29.62)	58 (23.02)		295 (51.22)	90 (48.91)	

†Data are presented as mean ± standard error or number (percentages), estimated mean and its standard error using sampling weight for complex sample

‡Using t-test of coefficient of estimates complex sample general linear model

§*P*-values < 0.05 indicate a statistically significant difference

Oral health behaviors showed significant associations with permanent decay in males, but not in females (*p*-value = 0.001). More specifically, male adolescents who had undergone a dental checkup within a year or brushed after every meal comprised a lower number of survey participants with permanent dental decay (dental checkup: 73.33% [No], 26.67% [Yes]; tooth brushing after every meal: 76.98% [No], 23.02% [Yes]).

**Fig. 1:**
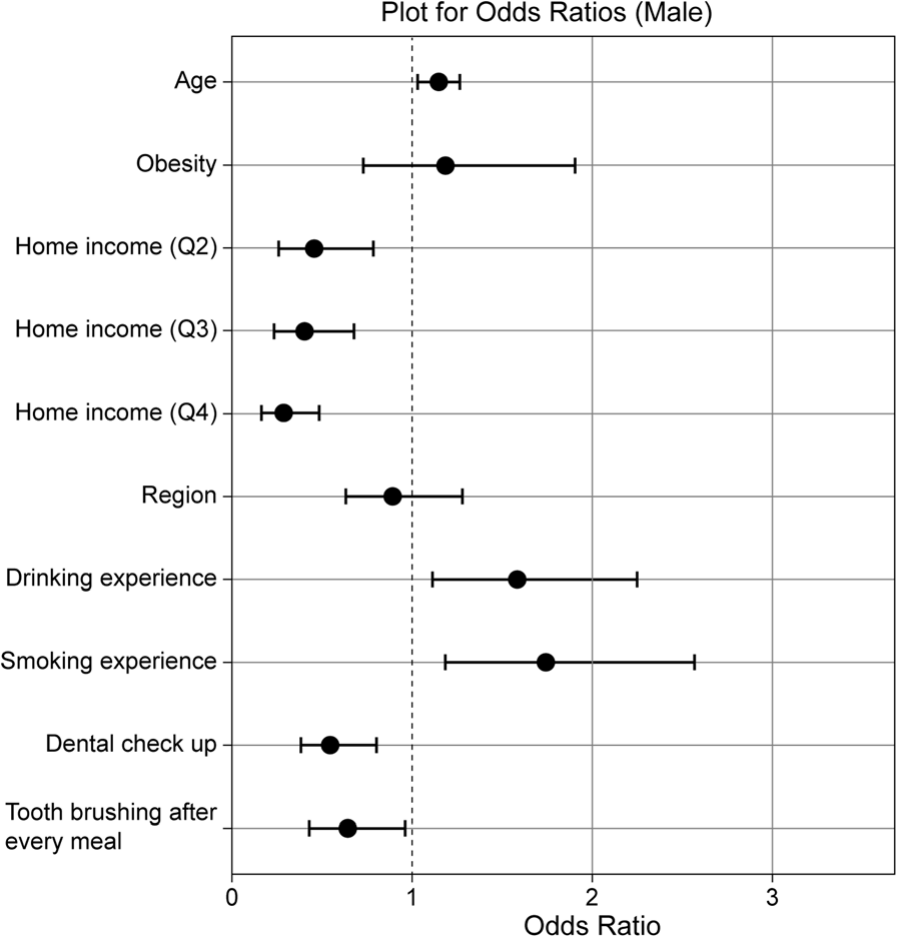
Univariate logistic regression analyses of obesity, socioeconomic status, and oral health behaviors in relation to incidence of dental cries on permanent teeth among male adolescents

**Fig. 2:**
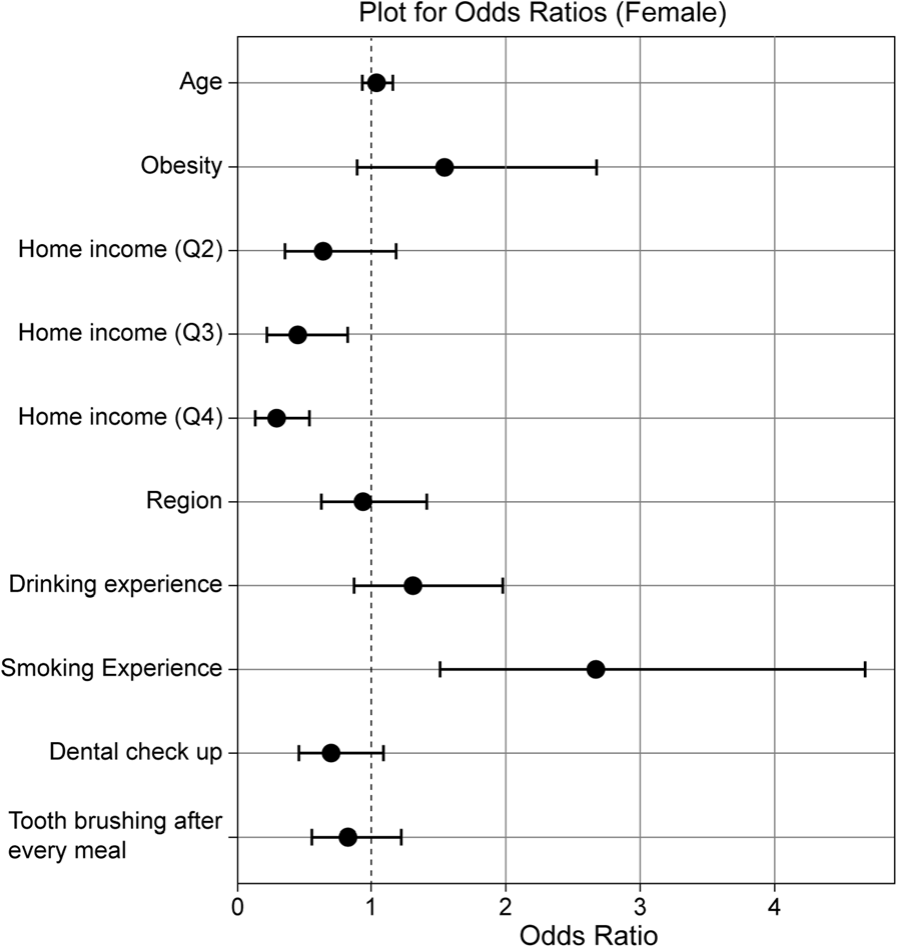
Univariate logistic regression analyses of obesity, socioeconomic status, and oral health behaviors in relation to incidence of dental cries on permanent teeth among female adolescents

Figures 1 and 2 present the odds ratios and 95% confidence intervals (CIs) from univariate logistic regression analyses of obesity, socioeconomic status, and oral health behaviors in relation to incidence of dental cries on permanent teeth for male and female adolescents separately. Both drinking and smoking experience were significantly associated with dental caries in male adolescents. However, only smoking experience was associated with dental caries in female adolescents.

Table 2 presents the odds ratios and 95% CIs from multiple logistic regression analyses of obesity, socioeconomic status, and oral health behaviors in relation to incidence of dental cries on permanent teeth for male and female adolescents separately. An association was detected between household income and permanent dental decay in male adolescents after all adjusting for variables. The odds ratios and 95% CIs thereof were 0.43 (0.24–0.76), 0.41 (0.24–0.70), and 0.28 (0.16–0.49) for the low-middle, middle-high, and high income groups, respectively. The association between smoking experience and dental cries on permanent teeth remained after adjustment for all variables in female adolescents (2.50 [1.36–4.58]). Although not significant, a similar trend was observed for male adolescents. Oral health behaviors, such as tooth brushing after every meal, showed associations with dental caries in only male adolescents. The odds ratio and CI for male survey participants who brushed after every meal was 0.63 (0.41–0.96). While our results suggested an association between oral health behaviors and dental cries on permanent teeth in Korean adolescents, we noted no association between obesity and dental cries on permanent teeth in either the male or female adolescent groups.

**Table 2:** Multivariate logistic regression analyses of obesity, socioeconomic status, and oral health behaviors on incidence of dental cries on permanent teeth

Variables	Dental caries incidence in male		Dental caries incidence in female	
	OR (95% CI)	*p*-value^†^	OR (95% CI)	*p*-value^†^
Age (years)	1.09 (0.95–1.24)	0.215	0.97 (0.84–1.12)	0.641
Obesity				
Non-obesity	1.00		1.00	
Obesity	1.37 (0.83–2.28)	0.221	1.52 (0.82–2.80)	0.179
Income (quartiles)				
Q1 (lowest)	1.00		1.00	
Q2	0.43 (0.24–0.76)	0.003	0.68 (0.37–1.27)	0.229
Q3	0.41 (0.24–0.70)	0.001	0.47 (0.25–0.90)	0.022
Q4 (highest)	0.28 (0.16–0.49)	<.001	0.31 (0.15–0.62)	0.001
Living area				
Rural	1.00		1.00	
Urban	0.77 (0.53–1.13)	0.183	1.12 (0.72–1.73)	0.624
Drinking experience				
No	1.00		1.00	
Yes	1.14 (0.71–1.83)	0.577	0.97 (0.57–1.62)	0.892
Smoking experience				
No	1.00		1.00	
Yes	1.54 (0.97–2.44)	0.065	2.50 (1.36–4.58)	0.003
Dental checkup within a year				
No	1.00		1.00	
Yes	0.67 (0.44–1.01)	0.055	0.67 (0.43–1.04)	0.075
Tooth brushing after every meal				
No	1.00		1.00	
Yes	0.63 (0.41–0.96)	0.033	0.98 (0.65–1.47)	0.921

†*P*-values < 0.05 indicate a statistically significant difference

## Discussion

This study was conducted to examine the associations among obesity, family socioeconomic status, oral health behaviors, and dental caries and to disclose possible differences in factors related with dental caries according to gender among Korean adolescents. Herein, we found that several socioeconomic characteristics and oral health behaviors were associated with dental caries and that these results differed significantly depending on gender among Korean adolescents. Nevertheless, we noted no association between obesity and dental caries. Therefore, our hypothesis that there would be a relationship between obesity and dental caries was not supported.

Previous studies have asserted that frequent consumption of mono and disaccharide sugars is the predominant cause of both obesity and dental caries [11, 12]. Soft drink consumption has also been shown to be a risk factor for obesity and dental caries among children and adolescents [13]. For these reasons, the Comprehensive Improvement Plans for School Food Service (2007~ 2011) and policies targeting the prohibition of beverage sales in school were enacted to prevent obesity caused by foods in Korea. Through these national efforts, adolescent obesity caused by sugar consumption and an unbalanced diet has been relatively controlled [14]. However, obesity caused by other factors, such as decreased physical activity and increased sedentary time, has been increasing [15]. As our study revealed that adolescent obesity is not related to dental caries, which are affected by sugary diets, and in light of the results of the studies above, taken altogether, this suggests that adolescent obesity in Korea is presently related more with a lack of exercise than diet.

Previous studies have explained that low socioeconomic status can be an impediment to oral health, because of the high cost of dental services, the low availability of dental insurance, poorer living conditions, less knowledge about the negative consequences of health-compromising behaviors, and greater psychological stress among adults [16–19]. Also, much of the literature has reported that socioeconomic status and smoking are correlated. Similarly, smoking experience among adolescents has been found to increase with lower parental socioeconomic status [19], further threatening the oral health of adolescents because of lots of hazardous substances in cigarettes. It is suggested that parents with good socioeconomic status are more likely to be interested in health education, including smoking cessation and abstinence. Like previous studies, the results of the present study support that higher household socioeconomic status could be a protective factor against dental caries in adolescents and that smoking experience is related to dental caries. Also, interestingly, we discovered that smoking experience was more strongly associated with dental caries in female adolescents than in male adolescents. According to Statistics Korea [20], the prevalence of smoking has decreased from 12.8% in 2009 to 7.8% in 2015 among adolescents. However, these rates are much higher than those in Australia (5.6%), Finland (5.0%), and Canada (1.9%), as well as other Asian countries, such as China (6.9%), Singapore (6.0%), and Japan (1.7%) [21]. Furthermore, smoking rates have increased significantly among female adolescents in Korea (3.2%) [21], a rate much higher than those in Canada (1.7%), China (2.2%), and Japan (1.1%) [20].Therefore, we suggest that it may be necessary to develop measures targeting dental health among adolescents of low socioeconomic status, so that they can live healthy lives even when they become adults. Moreover, oral health policies targeting female adolescents should stress the dental problems associated with smoking.

Oral health behaviors, such as an annual dental checkups and daily tooth brushing after every meal, are very important to reducing teeth plaque and calculus. Adolescent brushing habits, such as tooth brushing, interdental tooth brushing, and use of floss, are important, because they can affect oral health in adulthood. A previous study that examined the number of tooth brushings after every meal for 761 adolescents reported that 77.7% of the adolescent participants brushed their teeth after every meal [22]. In the present study, 27.7% of the male adolescents brushed their teeth after every meal, while 50.7% of the female adolescents brushed their teeth after every meal. Similarly, the percentage of female adolescents who underwent dental checkups within a year was higher than that in male adolescents. These results suggest better oral health behaviors among female adolescents of Korea than their male counterparts. According to a previous study that described the percentage of adolescents who brushed their teeth after lunch and factors related therewith, 40.6% of adolescents did not use floss, and 43.7% did not even know how to floss at all [23]. Ostberg [24] and Watt [25] asserted that oral health education in schools can expect only a short-term effect, because it does not consider individual characteristics. Therefore, attempts to strengthen oral hygiene education should be tailored to the individual’s knowledge and oral health condition.

As the data for this study were derived from the KNHANES, the largest representative survey in Korea, the present results are considered to accurately reflect the oral health statuses of and factors related to dental caries among Korean adolescents, particularly owing to the large sample size. However, as this was a cross-sectional study, a cause-and effect relationship between variables cannot be inferred. Therefore, we suggest the need for a school-based cohort study to clarify the factors affecting dental caries among adolescents.

## Conclusions

This study revealed associations between family socioeconomic status or oral health behavior and dental cries on permanent teeth in Korean male adolescents. In female adolescents, we noted associations between family socioeconomic status or smoking experience and dental caries. Unexpectedly, obesity was not found to be related to dental caries. In light of our results, we suggest that oral health interventions tailored to the individual gender may help in improving dental health among adolescents.

## Abbreviations

BMI: Body mass index
CI: Confidence interval
DMFT: Decayed, missing, and filled teeth index
KCDCP: Korea Centers for Disease Control and Prevention
KNHANES: Korea National Health and Nutrition Examination Survey
